# Intensification of the aerobic bioremediation of an actual site soil historically contaminated by polychlorinated biphenyls (PCBs) through bioaugmentation with a non acclimated, complex source of microorganisms

**DOI:** 10.1186/1475-2859-5-11

**Published:** 2006-03-20

**Authors:** Sara Di Toro, Giulio Zanaroli, Fabio Fava

**Affiliations:** 1 DICASM, Faculty of Engineering, Alma Mater Studiorum-University of Bologna, Viale Risorgimento 2, I-40136 Bologna, Italy

## Abstract

**Background:**

The biotreatability of actual-site polychlorinated biphenyl (PCB)-contaminated soils is often limited by their poor content of autochthonous pollutant-degrading microorganisms. In such cases, inoculation might be the solution for a successful bioremediation. Some pure and mixed cultures of characterized PCB degrading bacteria have been tested to this purpose. However, several failures have been recorded mostly due to the inability of inoculated microbes to compete with autochthonous microflora and to face the toxicity and the scarcity of nutrients occurring in the contaminated biotope. Complex microbial systems, such as compost or sludge, normally consisting of a large variety of robust microorganisms and essential nutrients, would have better chances to succeed in colonizing degraded contaminated soils. However, such sources of microorganisms have been poorly applied in soil bioremediation and in particular in the biotreatment of soil with PCBs. Thus, in this study the effects of Enzyveba, i.e. a consortium of non-adapted microorganisms developed from composted material, on the slurry- and solid-phase aerobic bioremediation of an actual-site, aged PCB-contaminated soil were studied.

**Results:**

A slow and only partial biodegradation of low-chlorinated biphenyls, along with a moderate depletion of initial soil ecotoxicity, were observed in the not-inoculated reactors. Enzyveba significantly increased the availability and the persistence of aerobic PCB- and chlorobenzoic acid-degrading cultivable bacteria in the bioreactors, in particular during the earlier phase of treatment. It also markedly enhanced PCB-biodegradation rate and extent (from 50 to 100%) as well as the final soil detoxification, in particular under slurry-phase conditions. Taken together, data obtained suggest that Enzyveba enhanced the biotreatability of the selected soil by providing exogenous bacteria and fungi able to remove inhibitory or toxic intermediates of PCB biodegradation and/or exogenous nutrients able to sustain microorganisms in charge for PCB mineralization.

**Conclusion:**

Enzyveba appears a promising agent for bioaugmenting actual-site PCB-polluted soils with a native low content of indigenous specialized microflora. This not only for its positive effects on the soil biotreatability but also for its availability on the market at a relatively low cost.

## Background

Polychlorinated biphenyls (PCBs) are xenobiotic compounds of great concern widely spread in the environment. PCBs occurring in soils can be partially biodegraded by consortia of aerobic PCB-cometabolizing bacteria and chlorobenzoic acid (CBA)-mineralizing bacteria [1-3]. The process can be intensified by amending the soils with biphenyl and oxygen [2-4] and performing the treatment under conditions able to provide a high degree of soil mixing and homogeneity [5,6]. However, the majority of studies reported in the literature has been performed on pristine soils amended with well-defined mixtures of PCBs and bacterial strains [2,3,6], whereas a few efforts have been made so far to intensify the bioremediation of real contaminated soils under field conditions or laboratory conditions resembling those applied on the large-scale remediation [3,4]. The bioremediation of actual-site aged PCB-contaminated soils is very often adversely affected by the low bioavailability of PCB and/or the scarcity of autochthonous pollutant-mineralizing microorganisms [4,7,8]. The bioavailability of pollutants (e.g. their occurrence in the soil water-phase, where microorganisms are located), might be improved by treating the soils in the presence of suitable PCB-"mobilizing" agents [9], also of biological origin [10-13]. The adverse effects due to the scarcity or limited competence of autochthonous pollutant-mineralizing microorganisms might be mitigated by bioaugmenting the soils with specialized exogenous microorganisms [7]. The basic premise for this intervention is the assumption that the metabolic capacities of microbial community already present in the biotope slated for cleanup will be increased by an exogenously enhanced genetic diversity, thus leading to a wider spectrum of productive biodegradation reactions [14-16]. The effectiveness of this approach has been tested in a number of PCB-spiked soils [1,6,17] but a little is still known about the possible role of bioaugmentation in the bioremediation of real contaminated soils. In general, pure cultures of PCB or CBA degrading bacteria [18-21], consortia of specialized bacteria [5,19,21-23], and genetically engineered bacteria able to avoid the accumulation of potentially toxic or dead-end intermediates of target pollutants [24] were applied for this purpose. Encouraging results have been sometimes obtained [18-21] but several failures have also been recorded [7,25,26]. The latter have been ascribed to barrier effects exerted by the soil ecological background (i.e. the diverse natural life forms living in communities within the soils), and to the limited availability of nutrients and/or the occurrence of toxic/inhibitory compounds in the inoculated biotopes [14-16,27]. Another approach poorly investigated so far is that of supplementing the contaminated soil with unspecified, naturally established complex consortia of microorganisms, such as those occurring in sludge, manure or compost [28]. These sources of microorganisms normally contain such a high diversity of microorganisms (bacteria, fungi, etc.) that the species necessary to biodegrade the pollutants and/or their metabolites may be present. Further, the addition of such a rich consortium of different microorganisms might result in the establishment of new and fruitfully interactions (at the catabolic and genetic level) between different microorganisms occurring at the augmented biotope and this in turn might result in an improved removal of pollutants [28]. These sources of microorganisms can also carry a variety of essential nutrients, that might strongly contribute to sustain survival and colonization of inoculated species in the biotope. Therefore, such sources of microorganisms appear of special interest for bioaugmenting complex biotopes like actual-site contaminated soils, generally characterized by a high toxicity, adverse pH and moisture content and a marked lack of nutrients [29]. On the other hand, current legislation regulating the management and restoration of contaminated sites in some European countries encourages the employment of some of such sources of microflora, with particular concern to those obtained from the microbial decomposition/stabilization of "adequate quality organic materials" from municipal wastes, in the biological restoration of contaminated soils and sites (see, as an example, the Italian D.M. No. 471/1999) [30]. Despite of this, only a little is currently known about the potential of such sources of microbial consortia in this field of soil bioremediation [8,28,29,31,32] and none of them have been tested so far in the bioremediation of actual-site PCB-contaminated soils. In the present work, the effects of a partially characterized consortium of microorganisms developed from the stabilization of high quality organic wastes on the aerobic bioremediation of an actual-site aged PCB-contaminated soil were studied in laboratory-scale slurry and solid-phase bioreactors. To the very best of our knowledge, this is the first work in which a not-adapted complex source of microorganisms is applied to bioaugment such a real PCB contaminated soil.

## Results

### Main features of S3 and of Enzyveba

S3 is an actual-site soil with over 10 years of storage in an Italian dump site. It was originally collected from the same site from which S1 soil employed in a previous study had also been obtained [13]. The content of C, N and P of sieved and air-dried S3 as well as its mechanical properties, pH and content of PCBs (determined qualitatively and quantitatively by using Aroclor1242 and Aroclor 1260 as standards), water and cultivable aerobic bacteria and fungi are given in Table 1. S3 was markedly contaminated by medium-highly chlorinated PCBs and endowed with a large amount of aerobic heterotrophic bacteria (10^7 ^CFU/g of air-dried soil) and with a low concentration of fungi (10^3 ^CFU/g of air dried soil). Conversely, it possessed indigenous aerobic bacteria capable of growing on biphenyl or on monochlorobenzoic acids (i.e., 2-, 3- and 4-chlorobenzoic acids) (CBAs) at concentrations close to the detection limit (10^2 ^CFU/g of dried soil) of the technique employed in the study. The water suspension of Enzyveba employed to bioaugment S3 displayed a pH of 7.7, and a significant amount of heterotrophic cultivable bacteria and fungi as well as a remarkable content of aerobic bacteria capable of growing on monochlorobenzoic acids (Table 2).

**Table 1:** Main features of soil. Main chemical, mechanical and microbiological properties of soil S3. Each value of biomass concentration is the average of duplicate analyses performed on a single sample of soil

	**Parameter values**
**Chemical characteristics**	
PCB concentration estimated by using Aroclor 1242 and 1260 as standards (mg/kg of dry soil)	920
Total Organic Carbon (g/kg)	19.5
Total Nitrogen (g/kg)	1.3
Total Phosphorous (g/kg)	0.7
Chloride ions (mg/l, in a 25% w/v soil suspension)	10.1
pH	7.2

**Mechanical characteristics**	
Moisture of the air-dried soil (g/kg)	7.3
Field capacity (% w/w)	20.3
Sandy fraction (0.053–2 mm) % w/w	90
Loamy fraction (0.002–0.0053mm) % w/w	9
Clay fraction (< 0.002 mm) %w/w	1

**Microbiological characteristics**	
Heterotrophic cultivable aerobic bacteria (CFU/g of dried soil)	4.88 × 10^7 ^± 7.00 × 10^5^
Aerobic bacteria growing on biphenyl (CFU/g of dried soil)	2.51 × 10^3 ^± 4.92 × 10^2^
Aerobic bacteria growing on CBA (CFU/g of dried soil)	< 10^2^
Total aerobic fungi (CFU/g of dried soil)	2.95 × 10^3 ^± 1.36 × 10^3^

**Table 2:** Main features of Enzyveba inoculum. Main chemical and microbiological properties of the Enzyveba suspension employed to inoculate the reactors. Each value of biomass concentration is the average of duplicate analyses performed on a single sample of inoculum

	**Parameter values**
**Chemical parameters**	
pH	7.7
Chloride ions (mg/l)	73

**Microbiological characteristics**	
Total heterotrophic cultivable aerobic bacteria (CFU/ml)	2.82 × 10^7 ^± 6.80 × 10^6^
Total aerobic bacteria growing on biphenyl (CFU/ml)	<10^2^
Total aerobic bacteria growing on CBA (CFU/ml)	4.50 × 10^4 ^± 5.00 × 10^3^
Total fungi (CFU/ml)	5.35 × 10^4 ^± 2.65 × 10^4^

### Biodegradation of PCBs in soil bioreactors

**Figure 1:**
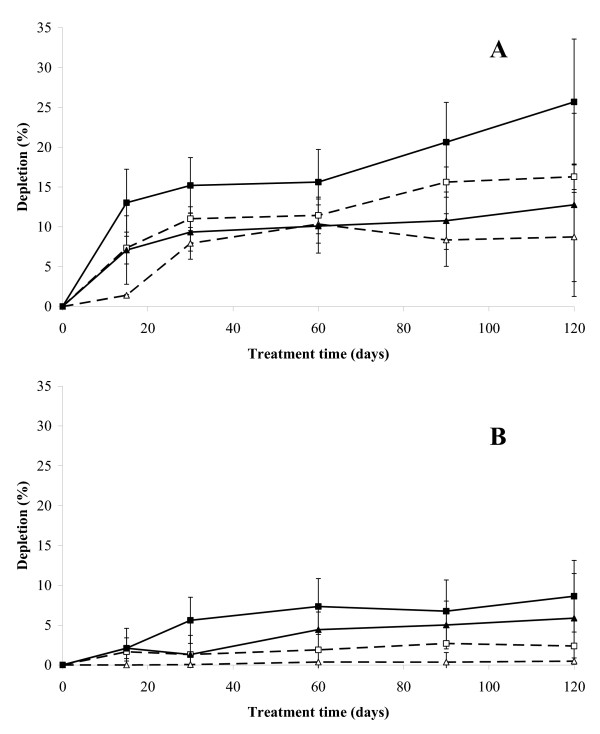
**Influence of the chlorination degree of PCBs on their biodegradation**. Depletion profiles of 2,4',5- and/or 2,4,4'-trichlorobiphenyl(s) (A) and of 2,2',3,3',4,5,5',6'-octachlorobiphenyl (B) in the slurry-phase reactors with () and without () Enzyveba and in the solid-phase reactors with () and without () Enzyveba. Each value is the average of duplicate analyses performed on soil samples collected from each of duplicate reactors (error bars represent standard deviation).

Aerobic conditions (with dissolved oxygen concentrations from 2 to 4 mg/l) and pH values close to 7.2 were observed in all developed soil bioreactors throughout the whole treatment (4 months). A detectable depletion of several soil PCBs was observed in all bioreactors at the end of the treatment (Table 3). However, the overall removal percentages decreased significantly by increasing the chlorination degree of congeners. As displayed by Figure 1, where the depletion profiles of a trichlorobiphenyl and those of an octachlorobiphenyl occurring in S3 are shown, PCB removal rates also decreased by increasing the chlorination degree of the molecules. In general, significantly faster and higher overall PCB depletions were observed in the slurry-phase reactors with respect to the solid-phase ones (Figure 1, Table 3). The presence of Enzyveba resulted in significantly improved rates (Figure 1) and final removal (by 75% and 135% under slurry and solid-phase conditions, respectively) of low-chlorinated biphenyls from the soil. Enzyveba addition also resulted in the significant depletion of some medium-highly chlorinated biphenyls not removed appreciably in the parallel not-inoculated reactors (Table 3).

**Table 3:** Soil PCBs and their depletion. PCBs detected in S3, their concentration and average depletions (in percentages ± standard deviation) after 120 days of treatment. Each value is the average of duplicate analyses performed on soil samples collected from each of duplicate reactors

**Target soil PCBs**	**Conc. (mg/kgss)**	**slurry phase**		**solid phase**		**Target soil PCBs**	**Conc. (mg/kgss)**	**slurry phase**		**solid phase**	
					
		**-**	**Enzyveba**	-	**Enzyveba**			**-**	**Enzyveba**	**-**	**Enzyveba**
2,6/2,2'	1.28	20.5 ± 3.6	22.9 ± 1.1	2.5 ± 0.9	6.8 ± 2.4	2,2',3,4',5',6/2,3',4,4',5	78.99	5.8 ± 9.5	18.7 ± 7.6	4.8 ± 4.3	8.4 ± 6.0
2,2',6	0.55	13.5 ± 0.7	16.9 ± 2.1	6.8 ± 0.3	7.2 ± 0.3	2,2',3,3',5,6	4.70	6.6 ± 4.7	11.5 ± 1.5	1.5 ± 2.0	3.9 ± 2.6
2,2',5/2,2',4/4,4'	8.76	16.1 ± 14.2	19.6 ± 7.8	8.7 ± 4.3	11.2 ± 7.2	2,2',3,3',4,6/2',3,3',4,5	3.34	5.3 ± 3.3	12.2 ± 5.9	1.3 ± 1.3	4.2 ± 2.0
2,3,6/2,3',6	0.55	11.2 ± 0.8	16.9 ± 3.5	7.3 ± 0.1	13.7 ± 0.3	2,2',3,4',5,5'	10.90	6.3 ± 1.8	12.3 ± 4.7	1.0 ± 0.3	5.3 ± 4.6
2,2',3/2,4',6	2.99	14.8 ± 4.8	18.6 ± 2.6	8.2 ± 1.0	13.2 ± 2.4	2,2',3,3',4,6'/2,2',4,4',5,5'/2,3,3',4,4'	174.11	5.8 ± 2.1	11.8 ± 7.7	0.6 ± 0.7	3.6 ± 3.5
2,3',5	1.37	5.5 ± 2.7	23.9 ± 4.2	3.7 ± 0.4	15.9 ± 1.5	2,2',3,4,5,5'/2,2',3,3',5,6,6'	30.00	4.4 ± 3.8	13.8 ± 6.3	0.7 ± 0.2	7.2 ± 6.1
2,4',5/2,4,4'	11.97	16.3 ± 1.6	25.7 ± 7.9	8.7 ± 5.6	12.8 ± 11.5	2,2',3,3',4,6,6'/2,2',3,4,4',5	7.63	5.2 ± 2.4	12.4 ± 2.9	0.7 ± 1.7	5.1 ± 2.6
2,3,3'/2',3,4/2,2',5,6'	2.23	16.7 ± 3.9	33.5 ± 11.3	7.4 ± 0.5	8.4 ± 1.7	2,2',3,4,4',5'/2,3,3',4,5,6/2,3,3',4,4',6	104.14	5.7 ± 3.8	11.5 ± 4.4	0.3 ± 1.7	2.9 ± 1.9
2,2',4,6'/2,3,4'	5.09	6.2 ± 3.3	21.5 ± 7.6	3.8 ± 2.2	12.3 ± 5.1	2,2',3,3',4,5/2,2',3,3',5,5',6	16.56	4.9 ± 1.0	10.6 ± 6.2	0.2 ± 0.2	5.3 ± 1.2
2,2',3,6	1.13	10.6 ± 1.4	24.3 ± 5.0	6.2 ± 0.2	10.4 ± 1.2	2,2',3,3',4,5',6	1.20	7.2 ± 1.1	9.9 ± 2.3	0.2 ± 0.2	4.8 ± 0.8
2,2',5,5'	4.70	9.5 ± 6.0	21.7 ± 3.1	6.1 ± 1.6	11.8 ± 3.9	2,2',3,4',5,5',6	31.27	0.7 ± 0.1	6.3 ± 2.4	0.7 ± 0.1	7.3 ± 4.2
2,2',4,5'	3.63	13.4 ± 4.5	21.3 ± 2.3	5.9 ± 1.2	8.9 ± 2.8	2,2',3,4,4',5',6	12.67	3.7 ± 1.5	4.7 ± 1.3	2.6 ± 2.0	3.4 ± 1.4
2,2',4,4'/2,2',4,5/2,4,4',6	1.96	8.7 ± 2.3	21.0 ± 11.8	5.7 ± 0.5	8.4 ± 1.5	2,2',3,3',4,4'	18.18	4.4 ± 1.8	2.4 ± 1.5	2.6 ± 2.2	4.0 ± 3.8
2,2',3,5'	3.56	12.6 ± 4.4	20.8 ± 2.3	5.8 ± 1.1	8.6 ± 2.7	2,2',3,4,5,5',6	7.50	1.9 ± 0.5	2.2 ± 0.4	1.7 ± 1.4	3.6 ± 5.0
3,4,4'/2,3,3',6/2,2',3,4'	2.00	9.5 ± 2.6	20.3 ± 1.4	3.4 ± 0.5	9.0 ± 1.6	2,2',3,3',4,5,6'	31.23	6.7 ± 3.5	8.7 ± 1.5	2.4 ± 1.8	5.6 ± 4.4
2,2',3,4/2,3,4',6	4.23	11.8 ± 5.2	20.3 ± 2.7	3.3 ± 1.2	8.6 ± 3.3	2,2',3,3',4',5,6	15.49	2.7 ± 1.0	4.1 ± 2.5	1.8 ± 0.9	4.6 ± 2.4
2,2',3,3'	1.18	9.3 ± 1.4	19.1 ± 3.5	2.7 ± 0.2	8.3 ± 1.0	2,2',3,3',5,5',6,6'/2,2',3,3',4,4',6/2,3,3',4,4',5	33.50	5.2 ± 2.8	5.2 ± 1.7	1.2 ± 0.5	3.5 ± 2.4
2,4,4',5	2.79	10.3 ± 3.5	20.1 ± 2.4	2.9 ± 0.8	11.4 ± 2.4	2,2',3,3',4,5,6/2,3,3',4,4',5'/2,2',3,3',4,5',6,6'	9.98	5.3 ± 3.0	6.3 ± 2.1	0.6 ± 0.3	3.9 ± 2.5
2,3',4',5	5.21	10.7 ± 6.8	19.7 ± 3.7	2.9 ± 1.7	4.5 ± 4.1	2,2',3,3',4,5,5'	6.30	6.1 ± 3.4	7.8 ± 2.1	0.6 ± 0.2	4.6 ± 4.4
2,3',4,4'/2,2',3,5',6	11.72	10.9 ± 4.7	20.6 ± 8.3	4.8 ± 4.4	11.3 ± 9.3	2,2',3,4,4',5,5'	64.49	4.4 ± 2.4	6.8 ± 2.7	0.1 ± 0.1	1.7 ± 1.3
2,3,4,4'/2,3,3',4'	4.72	8.6 ± 6.7	20.0 ± 4.2	2.7 ± 1.8	4.2 ± 3.8	2,3,3',4,4',5',6	1.87	9.8 ± 1.4	3.3 ± 1.7	8.5 ± 0.6	9.8 ± 1.2
2,2',3,5,5'	1.29	11.1 ± 1.6	17.3 ± 5.3	2.4 ± 0.6	5.0 ± 1.0	2,2',3,3',4,5,6,6'	2.31	6.8 ± 1.8	8.1 ± 4.8	0.5 ± 0.4	3.7 ± 1.5
2,2',3,3',6	1.54	10.2 ± 1.7	15.6 ± 6.4	2.4 ± 0.6	8.6 ± 1.0	2,2',3,3',4,4',5/2,3,3',4,4',5,6	62.89	8.0 ± 2.3	10.2 ± 2.2	1.3 ± 0.5	8.9 ± 2.9
2,2',3,4',5/2,2',4,5,5'	11.31	11.3 ± 6.6	17.4 ± 7.6	5.0 ± 3.5	7.5 ± 9.4	2,2',3,3',4,5,5',6'	8.63	2.4 ± 2.8	8.6 ± 4.5	0.5 ± 0.4	5.9 ± 5.6
2,2',4,4',5	1.69	9.6 ± 2.1	16.3 ± 6.2	2.1 ± 0.6	8.5 ± 1.1	2,2',3,4,4',5,5',6/2,2',3,3',4,4',5',6	12.06	6.9 ± 3.5	9.0 ± 6.4	-1.7 ± 3.3	4.9 ± 3.3
2,2',3',4,5	1.52	8.6 ± 1.8	16.0 ± 6.6	2.2 ± 0.6	7.4 ± 1.0	2,3,3',4,4',5,5'	1.65	0.8 ± 0.6	3.8 ± 0.5	4.2 ± 0.3	3.1 ± 1.3
2,2',3,4,5'	2.92	8.7 ± 3.6	15.0 ± 1.2	2.2 ± 1.3	7.7 ± 2.1	2,2',3,3',4,5,5',6,6'/2,2',3,3',4,4',5,6	6.22	2.1 ± 0.2	2.4 ± 1.5	0.4 ± 0.2	4.1 ± 3.3
2,2',3,3',6,6'	2.47	7.3 ± 2.7	10.7 ± 1.1	2.1 ± 1.0	4.2 ± 1.6	2,2',3,3',4,4',5,5'	11.28	2.7 ± 0.1	4.5 ± 2.7	1.5 ± 1.0	4.3 ± 1.5
						
3,3',4,4'/2,3,3',4',6	11.42	7.1 ± 4.9	12.1 ± 6.6	3.9 ± 4.2	7.8 ± 1.9						
2,2',3,5,5',6/2,2',3,3',4	21.29	5.6 ± 5.8	14.1 ± 3.1	2.3 ± 2.0	8.5 ± 7.3	**Total concentration**	**920.56 ± 3.76**				
2,2',3,3',5,6'	14.38	5.7 ± 5.4	13.5 ± 6.2	2.4 ± 1.6	7.1 ± 6.7	**Average removal**		**8.0 ± 0.5**	**14.0 ± 0.9**	**3.0 ± 0.3**	**7.1 ± 0.4**

**Figure 2:**
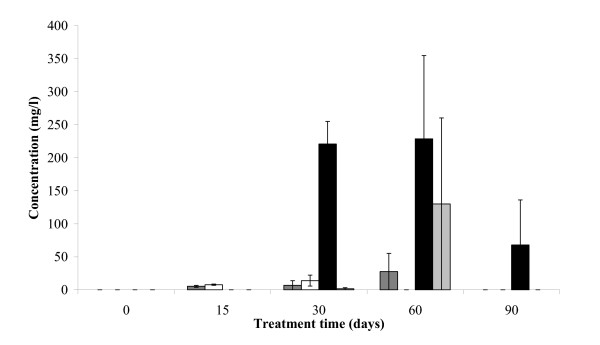
**Biotransformation of 2-chlorobenzoic acid in S3 reactors**. Transient accumulation of 2-chlorobenzoic acid in the slurry-phase reactors with () and without (□) Enzyveba and in the parallel solid-phase reactors with (■) and without () Enzyveba throughout the treatment. Each value is the average of duplicate analyses performed on soil samples collected from each of duplicate reactors (error bars represent standard deviation).

Several HPLC-Diode-Array detectable aromatic compounds having retention times and UV spectra comparable to those of CBAs were found to transiently accumulate in the reactors, in particular under solid-phase conditions. However, only one of them was characterized as CBA and ascribed to 2-chlorobenzoate, whereas none of the others co-eluted with any of the pure CBAs that were tested, i.e., 2-,3- and 4-monochlorobenzoic acids and 2,3-, 2,4-, 2,5-, 2,6-, 3,5-, and 3,4-dichlorobenzoic acids. A higher number of such metabolites were detected in the Enzyveba-supplemented reactors, where however their depletion was often faster than in the reference reactors (data not shown). As an example, the effects of Enzyveba on the fate of 2-CBA under slurry and solid-phase conditions are shown in Figure 2.

**Figure 3:**
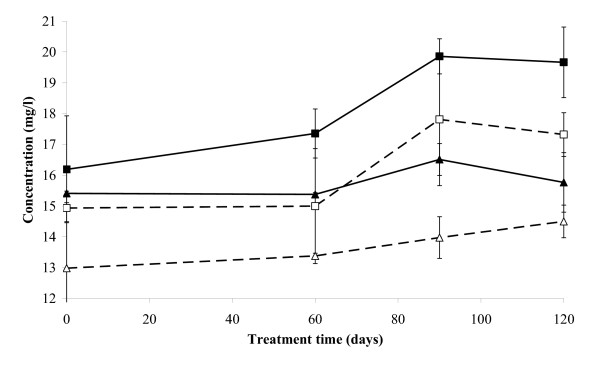
**Release of Chloride ions in S3 bioreactors**. Release of chloride ions in the slurry-phase reactors with () and without () Enzyveba and in the parallel solid-phase reactors with () and without () Enzyveba throughout the treatment. Each value is the average of analyses performed on soil samples collected from each of duplicate reactors (error bars represent standard deviation).

**Figure 4:**
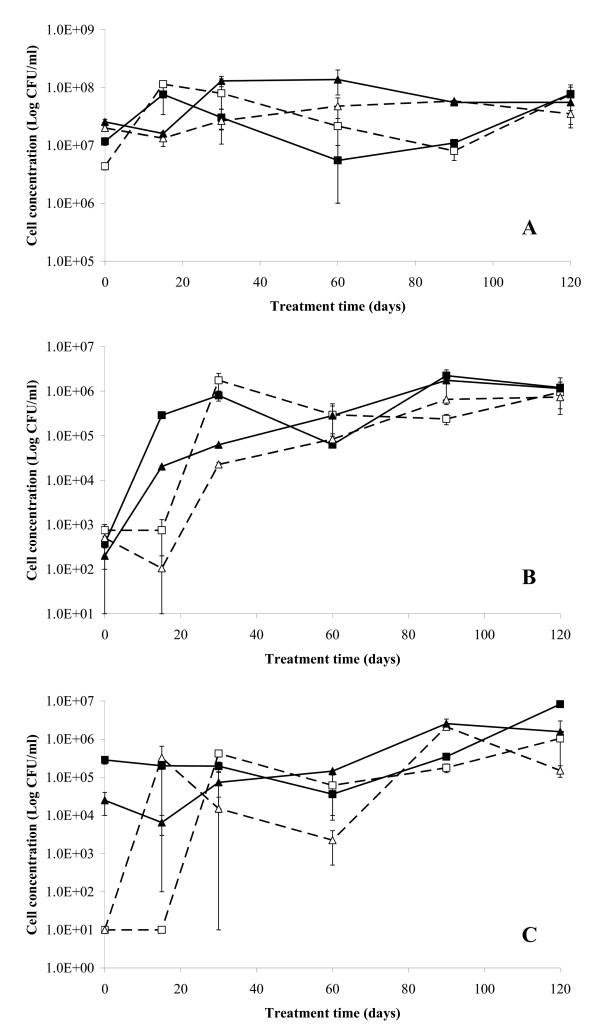
**Fate of aerobic heterotrophic and specialized cultivable bacteria in S3 bioreactors**. Changes in the concentration of the total aerobic cultivable heterotrophic bacteria (A) and of the total aerobic bacterial biomass able to grow on biphenyl (B) or on monochlorobenzoic acids (C) as a function of the treatment time in the slurry-phase reactors with () and without () Enzyveba and in the parallel solid-phase reactors with () and without () Enzyveba. Each value is the average of analyses performed on soil samples collected from each of duplicate reactors (error bars represent standard deviation).

**Figure 5:**
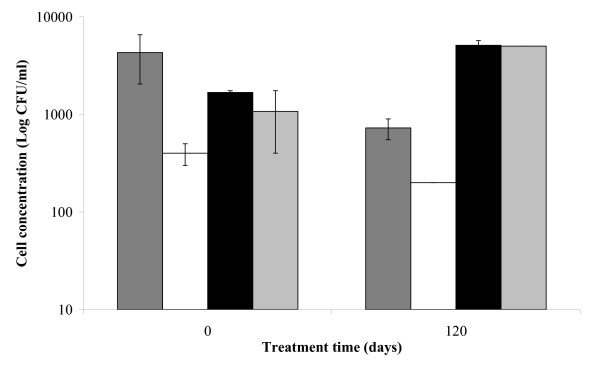
**Fungal biomass occurrence in S3 bioreactors**. Changes in the fungal counts as a function of the treatment time in the slurry-phase reactors with () and without (□) Enzyveba and in the parallel solid-phase reactors with (■) and without () Enzyveba. Each value is the average of analyses performed on soil samples collected from each of duplicate reactors (error bars represent standard deviation).

**Figure 6:**
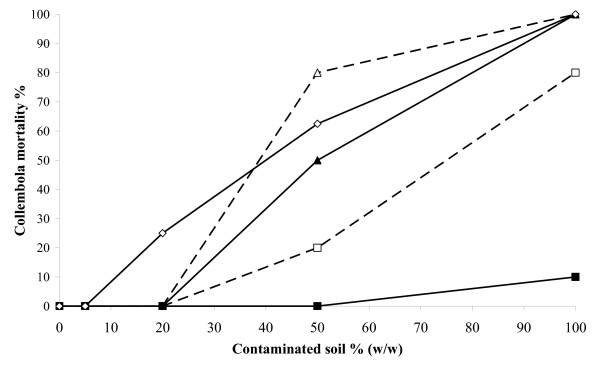
**Ecotoxicity of S3 before and at the end of the biological treatment**. Collembola mortality percentages versus the amount of contaminated soil occurring in the sample when it was assayed S3 before treatment () and S3 resulting from the four-months treatment in the slurry-phase reactors with () and without () Enzyveba and in the parallel solid-phase reactors with () and without () Enzyveba. Each value is the result of a single measurement on samples obtained by combining equal amounts of soil of the duplicate reactors.

A detectable release of Cl^- ^was observed in all S3 reactors throughout the treatment (Figure 3). The process was more rapid and extensive in the slurry-phase reactors than in the solid-phase ones. Enzyveba, which carried exogenous chloride ions (Table 2) by increasing the initial chloride concentration in the amended reactors by 1.825 ± 0.22 mg/l, significantly enhanced the process only under slurry-phase conditions (Figure 3). In general, significant changes in the concentration of the aerobic cultivable bacteria were observed in all S3 reactors throughout the experiment (Figure 4). At the beginning of treatment (3^rd ^day), the aerobic heterotrophic cultivable bacteria detected in the slurry- and solid-phase reactors were 4.35 × 10^6 ^± 6.00 × 10^5 ^and 2.00 × 10^7 ^± 9.50 × 10^5 ^CFU/ml, respectively. These values slightly increased throughout the treatment to reach final values that were comparable in the slurry and solid-phase reactors (Figure 4a). On the contrary, biphenyl- and monochlorobenzoic acid-metabolizing bacteria were poorly occurring in the two reactor systems (about 10^2^–10^3 ^CFU/ml and less than 10^2 ^CFU/ml, respectively) at the beginning of the experiment. However, both types of specialized cultivable biomass grew markedly in both reactor systems since the 15^th^–30^th ^day of incubation, by achieving final concentration values in the range 10^5^–10^6 ^CFU/ml (Figures 4b and 4c). A remarkable concentration of fungi was also detected in both reactor systems since the 3^rd ^day of incubation. Fungal biomass increased significantly throughout the treatment under solid-phase conditions, whereas it slightly decreased in the slurry-phase reactors (Figure 5). In the presence of Enzyveba, higher concentrations of heterotrophic, biphenyl- or CBA-growing bacterial biomass were generally observed in all reactors since the 3^rd ^day of incubation (Figure 4). Enzyveba also increased significantly the initial occurrence of fungi in both reactor systems. However, no marked differences in fungal biomass concentration were observed between inoculated and Enzyveba-free reactors at the end of the treatment (Figure 5). S3 exhibited a quite high original ecotoxicity. Relevant decreases of this parameter were observed at the end of the treatment, in particular under slurry-phase conditions (Figure 6). Enzyveba markedly enhanced the detoxification of the soil under both treatment conditions (Figure 6).

## Discussion

In this work, we investigated the effectiveness of an unusual bioaugmentation procedure based on the use of a complex consortium of not-adapted microorganisms, in the aerobic bioremediation of an actual-site PCB-contaminated soil. For this purpose, we selected the commercially available source of microorganisms Enzyveba, the heavily and historically actual site PCB-contaminated soil S3 and laboratory-scale treatment conditions, i.e., slurry- and solid-phase ones, that more closely resemble those more commonly employed in the large-scale bioremediation of chloroaromatic-contaminated soils [29]. Owing to the complexity of soil contamination and the absence of information on PCB metabolism by microorganisms of Enzyveba, an integrated analytical methodology, consisting of a combination of specific chemical, microbiological and ecotoxycological methods, was applied for a more efficient and reliable characterization of the impacts of inoculated microorganisms on the soil bioremediation and detoxification.

Low amounts of S3 original PCBs were removed in both reactor systems without Enzyveba at the end of the treatment (Table 3). Low chlorinated biphenyls were the S3 pollutants most rapidly and extensively removed (Figure 1 and Table 3). A transient accumulation of 2-CBA (Figure 2) and of other CBA-like intermediates as well as a remarkable release of chloride ions (Figure 3) and depletion of S3 initial ecotoxicity (Figure 6) were observed in the same bioreactors throughout the treatment. These findings along with the extensive proliferation of biphenyl- or CBA-metabolizing bacteria observed in the reactors (Figure 4) suggest that S3 PCBs were mostly removed through aerobic biodegradation and according to the common chlorobenzoic acids pathway [1]. Similar observations and conclusions have been reported by other authors who performed bioremediation studies on actual-site PCB contaminated soils [10,13,18,19,21,33]. Higher PCB biodegradation and dechlorination rates and extents (Figures 1 and 3; Table 3), along with higher soil ecotoxicity depletions (Figure 6) were observed under slurry-phase conditions than under solid-phase ones. This may be ascribed, according to other authors, to the higher degree of homogeneity and mass-transfer rates probably achieved under the former conditions [5,13,29,34-36].

However, unsatisfactory overall PCB removals (from 3 to 8% of the initial 920 mg/kg of total PCBs of S3) were generally achieved under both treatment conditions at the end of the experiment. This might be the consequence of a) the high complexity and degree of contamination of S3 (Table 1), b) the low bioavailability of PCBs which is common in soils like S3 with a quite long history of contamination [7,9] and c) the very low original soil content of autochthonous PCB- and CBA-degrading bacteria (Table 1). Data obtained from the Enzyveba-bioaugmented reactors suggest that the poor biotreatability of S3 was mainly due to the scarcity of indigenous specialized bacteria. Indeed, S3 supplementation with Enzyveba resulted in significant enhancements of rates and extents of soil PCB biodegradation and dechlorination (Table 3, Figures 1, 3) along with a significant intensification of soil detoxification (Figure 6). These effects can be ascribed to the larger availability of cultivable heterotrophic microorganisms and specialized bacteria observed in the amended bioreactors, in particular during the early stage of the treatment (Figures 4 and 5). Similar observations were reported by Fava and Bertin [21] and Fava et al. [5], who applied the three-membered PCB dechlorinating bacterial co-culture ECO3 to intensify the aerobic bioremediation of another actual site chronically PCB-contaminated soil using conventional and unconventional slurry-phase reactors. However, it is interesting to point out that Enzyveba enhanced the biodegradation of a broader number of PCBs, including several medium-high chlorinated congeners, with respect to ECO3 (Table 3) [21], and this might be ascribed to its marked content of fungi [37], often reported as broad spectrum PCB degrading microorganisms [38]. The higher availability and persistence of specialized cultivable bacteria in S3 inoculated bioreactors can be partially ascribed to the fact that Enzyveba carried exogenous CBA-degrading bacteria (Table 2), which might have contributed to the removal of intermediates of PCB biodegradation and/or other S3 toxic chemicals able to inhibit growth and activity of autochthonous PCB degrading bacteria. On the other hand, the beneficial effects on PCB degrading bacteria associated to the biological removal of inhibitory CBAs and other metabolites of PCB and CBA aerobic biodegradation have been already well documented in the literature [1-3,6,18]. Furthermore, it cannot be excluded that Enzyveba sustained autochthonous specialized microorganisms by also carrying exogenous nutrients or microbial surfactants able, in turn, to improve the availability of soil nutrients and/or pollutants in the soil-water phase [39]. Finally, it has to be taken into account that Enzyveba microflora and/or nutrients might have intensified S3 bioremediation and detoxification by also positively affecting the uncultivable autochthonous specialized microflora, which is reported as able to play a central role on the chemicals transformation in soils [8].

## Conclusion

Enzyveba was found capable of significantly enhancing the bioremediation and detoxification of the actual-site aged PCB contaminated soil employed in the study under slurry- and solid-phase conditions. This was ascribed to its ability to improve the availability and persistence of specialized biomass in the soil reactors. This finding should be confirmed through additional experiments directed to test Enzyveba on other actual-site soils contaminated by either PCBs and/or other poorly biodegradable chloro-aromatic pollutants, both at the laboratory scale and at the larger scale. Indeed, a successful inoculation in laboratory reactors does not guarantee success of the same strategy in the field. However, the results of this preliminary study suggest that Enzyveba is a promising bioaugmentation agent for the bioremediation of PCB-polluted soils, not only for its ability to intensify the process, but also for its availability on the market at relatively low cost. An additional advantage is that, because of its origin, its incorporation into contaminated soils is not only allowed but also encouraged by the current legislation regulating the remediation of contaminated sites in some European countries.

## Methods

### Chemicals

Chemicals used to prepare the minimal medium MMM and Tryptic Soy medium (TSA), as well as biphenyl, chlorobenzoic acids (CBAs), pure PCBs, PCB mixtures (used as analytical standards) and solvents for PCB extractions and liquid chromatography analyses are given elsewhere [10,21]. Malt extract, glucose and peptone, employed to prepare the medium used for fungal colonies counts, were provided by Biolife (Milan, Italy). The ultra-resi-analyzed water for Ion-Chromatography as well as solvents employed in the HPLC measurements and for PCBs and CBAs extraction were supplied by Mallinckrodt-Baker (Phillipsburg, NJ, USA). The sources of the *Folsomia candida *(Collembola) and of the material used in the ecotoxicological measurements were already reported [21].

### Source of microorganisms

Enzyveba is a complex and stable consortium of prokaryotic and eukaryotic microorganisms patented and commercialized by Marcopolo Engineering SpA (Cuneo, Italy) as bioactivator for landfills, composting facilities and wastewater treatment plants. It was developed through a series of enrichments conducted under solid-state conditions on a variety of organic matter-rich vegetal and animal wastes for more than 20 years. It has been characterized through conventional and molecular procedures; according to the data available, it consists of a large variety of aerobic and anaerobic bacteria [40,41] and fungi (mostly ascomycetes and, to less extent, basidiomycetes) [37,42]. Enzyveba was supplied to S3 as a water suspension prepared by dispersing 45 g of Enzyveba dried-powder in 150 ml of distilled water then incubated at 35°C on a rotary shaker operating at 100 rpm for 1 day.

### Soil manipulations, reactors preparation and monitoring

S3 (3 kg) was homogenised, air-dried, sieved through a 0.2 cm sieve and analysed for its content of organic carbon (C), total nitrogen (N), total phosphorous (P), field capacity (that is the maximum amount of water that a soil can retain under given environmental conditions), moisture and pH according to Fava et al. [10], as well as for its mechanical properties as previously reported [5]. S3 was also analysed for its content of heterotrophic aerobic cultivable bacteria, fungi and PCB- and CBA-degrading aerobic cultivable bacteria. S3 was then supplemented with biphenyl (4 g/kg) and homogenized. It was divided in two portions of 1.5 kg each; one was amended with 150 ml of distilled water (to prepare the control S3 soil) and the other one with 150 ml of Enzyveba water suspension (to prepare the bioaugmented soil). A set of 4 slurry-phase reactors and a set of 4 solid-phase reactors (2 control reactors and 2 bioaugmented ones per set) were developed by, respectively, suspending 175 g of soil in 700 ml of water (soil suspension at 25% w/v) and dispensing 300 g of soil inside 1.0 Liter-baffled bottles then partially closed with Teflon liner-screw caps. Slurry reactors were placed on a rotary shaker working at 200 rpm, whereas the solid-phase ones were incubated statically but weekly mixed through repeated inversions. All reactors were kept at 20 ± 2°C in the dark and sampled after 3, 15, 30, 60, 90 and 120 days of incubation. Samples collected from slurry-phase reactors (80 ml) were subjected to centrifugation at 3,000 × *g*. The soil phase (about 20 g of wet soil) was divided in two portions, then subjected to a) solvent extraction (8 g of wet soil) followed by GC analyses (for PCBs) of the obtained organic extracts, and b) air-drying (12 g of wet soil were allowed to dry at room temperature under a hood for 3 days) followed by ecotoxicity analyses. The water phase was subjected to analysis of the concentration of a) CBAs (and other aromatic polar metabolites), b) chloride ions, c) heterotrophic, PCB-co-metabolising and CBA-degrading cultivable aerobic bacteria and d) total fungi. The soil treated under solid-phase conditions was managed as follows: 30 g of soil collected from the reactors were suspended in distilled water (120 ml) to have a 25% (w/v) soil suspension which was then shaken at 200 rpm and 20 ± 2°C for 2 days. The resulting slurries were centrifuged and the obtained phases analysed for PCBs, CBAs, chloride ions, cultivable aerobic bacterial biomass and fungi and ecotoxicity as reported above for the soil samples obtained from the slurry-phase reactors.

### Extraction and analytical procedures

PCBs were extracted from the soil-phase by using a mixture of hexane:acetone (1:1) in a Pressurized Fluid Extraction system (Dionex Corporation, Sunnyvale, CA, USA) operating at 140 atm and 100°C according to the procedure US-EPA-SW-846, Method 3545A. CBAs were batch extracted from the water phases by using diethyl-ether [11]. The qualitative and quantitative analysis of PCBs occurring in the organic extracts was performed with a gas chromatograph (5890 series II), equipped with a HP-5 capillary column (30 m by 0.25 mm) and an electron capture detector (ECD) (Hewlett-Packard Co., Palo Alto, CA, USA) according to the procedures described by Fava et al. [10]. The depletion of each selected PCB was calculated from the average of two GC analyses of samples collected from the two parallel identical slurry or solid phase reactors at each given sampling time. HPLC analysis of the diethyl ether extracts containing CBAs and other aromatic compounds was performed with a Beckman HPLC system equipped with a Beckman ultrasphere 4.6 × 250 mm ODS column (particle diameter = 5 *μ*m) and a 168 System Gold Diode Array detector operating at 235 and 254 nm (Beckman Instruments, Fullerton, CA, USA) [11]. The concentration of CBAs was calculated from the average of two HPLC measurements on samples collected from the two parallel identical slurry or solid phase reactors at each given sampling time. The concentration of Cl^- ^was measured by using a Dionex DX-120 IC system equipped with an IonPac AS14 4x250 mm column, a conductivity detector combined to a ASRS-Ultra conductivity suppressor system (Dionex Corporation, Sunnyvale, CA, USA). The eluent was a solution of 3.5 mM Na_2_CO_3 _and 1.0 mM NaHCO_3 _prepared in ultra-resi-analyzed water; the flow rate was 1.2 ml/min and the injection volume was 20 μl. Chloride ion concentrations were determined by performing the average of the results obtained from the analyses of samples collected from the two parallel identical slurry or solid phase reactors at each given sampling time. The concentration of the aerobic heterotrophic cultivable bacterial biomass and that of the biphenyl- or CBA-growing aerobic cultivable bacteria was determined by the plate-counting technique described by Fava and Di Gioia [11]. Fungal biomass occurring in the same reactors was counted at the beginning and the end of the experiment by using the same technique and agar plates of a medium consisting of (in g/l): malt extract, 20; glucose, 20; and peptone, 2. Bacterial and fungal biomass concentrations were calculated by running the average of the results of colonies counting performed on samples collected from the two parallel identical slurry or solid phase reactors at each given sampling time. Ecotoxicity measurements were performed on S3 air-dried samples by using the *Folsomia candida *(Collembola) acute toxicity test as described by Fava and Bertin [21]. Ecotoxicity measurements were carried out on samples obtained by combining equal amounts of soil sampled from the two parallel identical slurry or solid phase reactors at each given sampling time. Dissolved O_2 _concentration and pH of soil were measured on slurries (sampled from the slurry reactors or those prepared from the soil collected from the solid-phase ones) with an O_2 _selective electrode (97–08 model) and a pH probe (81–04 model), respectively (Orion Research Inc., Beverly, MA, USA).

## Competing interests

The author(s) declare that they have no competing interests.

## Authors' contributions

SDT carried out the experimental work described in the paper, GZ took part in some of the experiments described and FF coordinated the research as well as the manuscript preparation. All authors read and approved the final manuscript.

## References

[B1] Focht DD (1995). Strategies for the improvement of aerobic metabolism of polychlorinated biphenyls. Curr Opinion Biotechnol.

[B2] Ohtusubo Y, Kudo T, Tsuda M, Nagata Y (2004). Strategies for bioremediation of polychlorinated biphenyls. Appl Microbiol Biotechnol.

[B3] Pieper DH (2005). Aerobic degradation of polychlorinated biphenyls. Appl Microbiol Biotechnol.

[B4] Robinson GK, Lenn MJ (1994). The bioremediation of polychlorinated biphenyls (PCBs): problems and perspectives. Biotechnol Gen Engineer Rev.

[B5] Fava F, Di Gioia D, Marchetti L (2000). Role of the reactor configuration in the biological detoxification of a dump site polychlorobiphenyl-contaminated soil in lab slurry phase conditions. Appl Microbiol Biotechnol.

[B6] Abraham WR, Nogales B, Golyshin PN, Pieper DH, Timmis KN (2002). Polychlorinated biphenyl-degrading microbial communities in soils and sediments. Curr Opin Microbiol.

[B7] Providenti MA, Lee H, Trevors JT (1993). Selected factors limiting the microbial degradation of recalcitrant compounds. J Ind Microbiol.

[B8] Alexander M (1999). Biodegradation and bioremediation.

[B9] Volkering F, Breure AM, Rulkens WH (1998). Microbiological aspects of surfactant use for biological soil remediation. Biodegradation.

[B10] Fava F, Di Gioia D, Marchetti L (1998). Cyclodextrin effects on the *ex-situ *bioremediation of a chronically polychlorinated biphenyl-contaminated soil. Biotechnol Bioeng.

[B11] Fava F, Di Gioia D (2001). Soya lecithin effects on the aerobic biodegradation of polychlorinated biphenyls in an artificially-contaminated soil. Biotechnol Bioeng.

[B12] Fava F, Piccolo A (2002). Effects of humic substances on the bioavailability and aerobic biodegradation of polychlorinated biphenyls in a model soil. Biotechnol Bioeng.

[B13] Fava F, Bertin L, Fedi S, Zannoni D (2003). Methyl-beta-cyclodextrin-enhanced solubilization and aerobic biodegradation of polychlorinated biphenyls in two aged-contaminated soils. Biotechnol Bioeng.

[B14] Goldstein RM, Mallory LM, Alexander M (1985). Reasons for possible failure of inoculation to enhance biodegradation. Appl Environ Microbiol.

[B15] Pritchard PH (1992). Use of inoculation in bioremediation. Curr Opinion Biotechnol.

[B16] Vogel TM, Walter MV, Hurst CJ, Crawford RL, Knudsen GR, Mclnerney MJ, Stetzenbach LD. (2001). Bioaugmentation. Manual of Environmental Microbiology.

[B17] Top EM, Springael D, Boon N (2002). Catabolic mobile genetic elements and their potential use in bioaugmentation of polluted soils and waters. FEMS Microbiol Ecol.

[B18] Focht DD, Searles DB, Koh SC (1996). Genetic exchange in soil between introduced chlorobenzoate degraders and indigenous biphenyl degraders. Appl Environ Microbiol.

[B19] Abramowicz DA, Brown JF, Harkness MR, O'Donnell MK, Hickey RF, Smith G. (1996). In situ anaerobic PCB dechlorination and aerobic PCB biodegradation in Hudson River sediments. Biotechnology in Industrial waste treatment and bioremediation.

[B20] Evans BE, Dudley CA, Klasson KT (1996). Sequential anaerobic-aerobic biodegradation of PCBs in soil slurry microcosms. Appl Biochem Biotechnol.

[B21] Fava F, Bertin L (1999). Use of exogenous specialized bacteria in the biological detoxification of a dump site-polychlorinated biphenyl-contaminated soil in slurry phase conditions. Biotechnol Bioeng.

[B22] Klasson KT, Barton JW, Evans BS, Reeves ME (1996). Reductive microbial dechlorination of indigenous polychlorinated biphenyls in soil using a sediment-free inoculum. Biotechnol Prog.

[B23] Rojas-Avelizapa NG, Martinez-Cruz J, Zermeno-Eguia Lis JA, Rodriguez-Vazquez R (2003). Levels of polychlorinated biphenyls in Mexican soils and their biodegradation using bioaugmentation. Bull Environ Contam Toxicol.

[B24] Reineke W (1998). Development of hybrid strains for the mineralization of chloroaromatics by patchwork assembly. Annu Rev Microbiol.

[B25] Morgan P, Watkinson RJ (1989). Microbiological methods for cleanup of soil and ground water contaminated with halogenated organic compounds. FEMS Microbiol Rev.

[B26] Harkness MR, McDermott JB, Abramowicz DA, Salvo JJ, Flanagan WP, Stephens ML, Mondello FJ, May RJ, Lobos JH, Carrol KM, Brennan MJ, Bracco AA, Fish KM, Warner GL, Wilson PR, Dietrich DK, Lin DT, Morgan CB, Gately WL (1993). In situ stimulation of aerobic PCB biodegradation in Hudson river sediments. Science.

[B27] Van Veen JA, Van Overbeek LS, Van Elsas JD (1997). Fate and activity of microorganisms introduced into soil. Microbiol Mol Biol Rev.

[B28] Dejonghe W, Boon N, Seghers D, Top EM, Verstraete W (2001). Bioaugmentation of soils by increasing microbial richness: missing links. Environ Microbiol.

[B29] Cookson JT, Cookson JT (1995). Solid- and slurry-phase bioremediation. Bioremediation engineering, design and application.

[B30] Italian Ministry of Environment Technical Regulation D.M. No. 471/1999 on containment, remediation and environmental restoration of contaminated soils. Ordinary Annex of the Official Italian Gazette no 293; 12151999.

[B31] Kastner M, Mahro B (1996). Microbial degradation of polycyclic aromatic hydrocarbons in soils affected by the organic matrix of compost. Appl Microbiol Biotechnol.

[B32] Hupe K, Lüth JC, Heerenklage J, Stegmann R (1996). Enhancement of the biological degradation of soils contaminated with oil by the addition of compost. Acta Biotechnol.

[B33] Fava F, Di Gioia D (1998). Effects of Triton X-100 and Quillaya Saponin on the *ex-situ *bioremediation of a chronically polychlorobiphenyl-contaminated soil. Appl Microbiol Biotechnol.

[B34] Fava F, Ciccotosto FV (2002). Effects of Randomly Methylated-*β *-Cyclodextrins (RAMEB) on the bioavailability and aerobic biodegradation of polychlorinated biphenyls in three pristine soils spiked with a transformer oil. Appl Microbiol Biotechnol.

[B35] Fava F, Berselli S, Conte P, Piccolo A, Marchetti L (2004). Effects of humic substances and soya lecithin on the aerobic bioremediation of a soil historically contaminated by polycyclic aromatic hydrocarbons (PAHs). Biotechnol Bioeng.

[B36] Christodoulatos C, Koutsospyros A, Lewandowski GA, DeFilippi LJ. (1998). Bioslurry reactors. Biological treatment of hazardous wastes.

[B37] Anastasi A, Varese GC, Filipello Marchisio V (2005). Isolation and identification of fungal communities in compost and vermicompost. Mycologia.

[B38] Beaudette LA, Davies S, Fedorak PM, Ward OP, Pickard MA (1998). Comparison of gas chromatography and mineralization experiments for measuring loss of selected polychlorinated biphenyl congeners in cultures of white rot fungi. Appl Environ Microbiol.

[B39] Christofi N, Ivshina IB (2002). Microbial surfactants and their use in field studies of soil remediation. J Appl Microbiol.

[B40] Pietronave S, Fracchia L, Martinotti MG (2004). Influence of biotic and abiotic factors on human pathogens in a finished compost. Wat Res.

[B41] Fracchia L, Dohrmann AB, Martinotti MG, Tebbe CC Bacterial diversity in a finished compost and vermicompost – Differences revealed by cultivation-independent analyses of PCR-amplified 16S rRNA genes. Appl Microbiol Biotechnol.

[B42] Anastasi A, Varese GC, Voyron S, Scannerini S, Filipello Marchisio V (2004). Characterization of fungal biodiversity in compost and vermicompost. Compost Science & Utilization.

